# Expanding the impact of a longstanding Canadian cardiac registry through data linkage: challenges and opportunities

**DOI:** 10.23889/ijpds.v3i3.441

**Published:** 2018-11-12

**Authors:** Danielle A Southern, Matthew T James, Stephen B Wilton, Lawrence DeKoning, Hude Quan, Merril L Knudtson, William A Ghali

**Affiliations:** 1 Department of Community Health Sciences, O’Brien Institute for Public Health, University of Calgary, 2500 University Dr NW, Calgary, Alberta T2N 1N4, Canada; 2 Cumming School of Medicine, University of Calgary, 2500 University Dr NW, Calgary, Alberta T2N 1N4, Canada; 3 Libin Cardiovascular Institute of Alberta, University of Calgary, 2500 University Dr NW, Calgary, Alberta T2N 1N4, Canada; 4 Calgary Laboratory Services, 3535 Research Road NW, Calgary, AB, T2L 2K8; 5 Department of Pathology and Laboratory Medicine, Cumming School of Medicine, University of Calgary, 2500 University Dr NW, Calgary, Alberta T2N 1N4, Canada; 6 Department of Paediatrics, Alberta Children's Hospital, 2888 Shaganappi Tr NW, Calgary, Alberta, T3B 6A8, Canada

## Abstract

The Alberta Provincial Project for Outcome Assessment in Coronary Heart Disease (APPROACH) began as a province-wide inception cohort of all adult Alberta residents undergoing cardiac catheterization for ischemic heart disease. Strengths of the APPROACH initiative include the prospective collection of detailed clinical, procedural, and treatment information, measured at point-of-care. While this aspect of APPROACH provides data users with several advantages over use of typical administrative data, the ability to link APPROACH with data from multiple other sources has provided several unique opportunities to measure cardiovascular care and outcomes. As of June 2018, clinical information has been collected by APPROACH on over 240,000 adult Alberta residents. Linkage of this rich clinical data to administrative health data (eg. Vital statistics, hospitalizations, ambulatory events, prescription medications), secondary use clinical data (e.g. laboratory, ECG, rehabilitation, EMR, imaging) and other data sources (eg. Geospatial, crime data, meteorological) allows better study of the determinants of a patient’s health trajectory. This paper describes applied examples of work that has leveraged the potential of linking several datasets with the APPROACH registry.

The Alberta Provincial Project for Outcome Assessment in Coronary Heart Disease (APPROACH) began as a province-wide inception cohort of all adult Alberta residents undergoing cardiac catheterization for ischemic heart disease [1]. Data collection was initiated in January 1995 to facilitate study of provincial outcomes of care and facilitate quality improvement for patients with coronary artery disease in Alberta. Over its more than two decades of existence, the APPROACH initiative has grown in number of enrolled patients, number of participating regions, and in the scope of cardiovascular care and outcomes included. Since 2004, APPROACH has included data for patients hospitalized to a cardiac service for a wide range of both ischemic and non-ischemic cardiovascular conditions [2]. As of June 2018, clinical information has been collected by APPROACH on over 240,000 adult Alberta residents. Use of the APPROACH platform has also expanded across Canada and now facilitates the measurement and reporting of cardiovascular care across more than 18 major centres in 8 provinces. The Canadian Institutes of Health Research selected APPROACH as one of six Top Achievements in Health Research in 2009-2010 [3].

Strengths of the APPROACH initiative include the prospective collection of detailed clinical, procedural, and cardiovascular treatment information, measured at point-of-care. While this aspect of APPROACH provides data users with several advantages over the use of typical administrative data, the ability to link APPROACH with data from multiple other sources has provided several unique opportunities to measure cardiovascular care and outcomes. This paper describes applied examples of work that has leveraged the potential of linking several external datasets with the APPROACH registry.

## Global and Specific Objectives of APPROACH

The APPROACH initiative was developed in response to a need for ongoing assessment of the processes and outcomes of care for patients with coronary artery disease. Within the global objective of studying processes and outcomes, a number of specific objectives were identified when APPROACH started [1]. These objectives include the following:

- To monitor revascularization decisions after cardiac catheterization;
- To describe and monitor uptake of new therapies (eg, new revascularization techniques, new medications);
- To assess the impact of planned and unplanned treatment delays before revascularization procedures;
- To study both short term and long term risk-adjusted clinical outcomes (eg death, repeat admissions) after cardiac catheterization;
- To assess long term costs of post-catheterization treatment strategies;
- To report on short- and long-term quality of life outcomes;
- To foster collaborative interaction with other cardiovascular outcome assessment projects, both within and outside of Canada; and
- To create a permanent database tool for continuous quality measurement useful to both province-wide studies and to individual hospitals seeking to meet hospital accreditation requirements.

More recently the objectives of the APPROACH initiative have evolved to also include:

- To move the inception point for inclusion into the registry to other points of care entry in a patient’s history, from the point of cardiac catheterization to the point of admission to hospitals throughout Southern Alberta with a heart disease diagnosis.
- To leverage the computerized data collected in the APPROACH system to evaluate the clinical impact of point-of-care clinical decision support tools.
- To support operational decision-making by providing an interactive reporting package.

## Salient Features of APPROACH

The APPROACH database contains detailed clinical information on adult patients with known or suspected coronary artery disease (CAD) who are admitted to a cardiac ward and/or undergo invasive cardiac procedures. Patients in APPROACH are followed longitudinally after cardiac catheterization, thus allowing for assessment of subsequent procedure use (i.e. percutaneous coronary intervention (PCI) or coronary artery bypass graft surgery (CABG)), as well as outcomes such as mortality and quality of life. Data collection is ongoing, and as is typical in prospective data registries, there are occasionally data fields that are not completed in the data collection process.

APPROACH is designed to capture all patients undergoing cardiac catheterization in Alberta. This has been accomplished by integrating data entry within clinical workflow: data are entered by clinical staff at the time of service provision, and populate the relevant procedure reports as well as the research database. All 3 sites in Alberta that provide catheterization to the provincial population use the APPROACH database. This allows the processes and outcomes of cardiac procedures to be studied at a population level, rather than in a convenience sample of selected patients, such as those from a single referral centre or those receiving a single treatment where findings may not be applicable to the total population. Therefore, unbiased population-based information is obtained on the large number of patients undergoing cardiac procedures in Alberta. This system reduces the costs of data collection, while also facilitating research activity at the population level.

Information is collected regarding the patient’s procedure as well as his/her comorbidities. Patients have also been invited to provide follow up information on their experience, generic and disease-specific quality of life, and outcomes at baseline and 1, 3 and 5 years after cardiac catheterization. This allows researchers to investigate the path and impact of care on patient health trajectory, while also allowing researchers to identify patients who provide consent to participate in future ancillary research studies. The APPROACH research data currently includes 240,000 patients, of which 186,869 adult patients (approx. 29% women) received cardiac catheterization in the province of Alberta between April 1 1995 and March 31 2017, with a follow-up time for survival of over 22 person years (for the earliest patients enrolled).

A unique feature of the APPROACH database is the data collection process. After a patient is referred for a catheterization procedure, they are first registered into the system, which allows tracking of wait times. Prior to procedures, demographic and clinical information is collected by the relevant clinicians, including nursing staff, physicians, and other cardiac catheterization laboratory technicians. At this stage, patient comorbidities and past medical history are collected both directly from patients as well as from medical records. Following completion of cardiac procedures, a sophisticated and unique tool (Coronary Artery Reporting and Archiving Tool (CARAT)) is used to record granular coronary artery disease information and treatment information entered at point of care by clinicians [4]. CARAT is a mandated clinical reporting tool in Alberta and provides a visual representation of severity of disease and treatment that is linked to electronic medical records to directly enter the patient’s chart and immediately communicate diagnostic and treatment information for patients and care providers. Detailed information regarding test and procedure results is directly recorded by clinicians and other laboratory personnel [1]. Following discharge, patients are given the opportunity to consent to be contacted in future follow-up to participate in subsequent research studies as well as contribute patient-reported outcome and experience measures.

APPROACH is structured as a relational database with fields pre-defined rather than dependent on free text. Common data definitions are used throughout, based on alignment with Canadian Cardiovascular Society (CCS) data definitions and the National Cardiovascular Data Registry (NCDR) cardiac catheterization data definitions [5, 6]. Figure 1 shows the data modules that have been created within APPROACH for data collection.

The data collection process in APPROACH provides two major advantages. First, data must be entered according to consistent criteria outlined by data definition documentation, unlike administrative data, where the documentation is variable and depends on the discretion of the physician or the coding practices of a particular hospital. APPROACH uses prospective data collection performed by nurses and physicians for patient characteristics, comorbidities, and details of management during the index hospitalization. Second, the direct collection of data in the clinical setting reduces the chance of error due to data translation. This distinguishes APPROACH data from administrative data, where clinicians typically do not enter data directly. Rather, it is usually health record coders who record information from medical charts in administrative data systems.

Since 2004, a cohort of patients have been captured in the APPROACH database when they are admitted to a cardiac ward in acute care hospitals in Southern Alberta. The diagnosis of acute coronary syndrome (ACS) and its subcategorization as unstable angina, non-ST elevation myocardial infarction (NSTEMI), and ST elevation myocardial infarction (STEMI), are entered at the time of discharge, and represent the physician’s interpretation of all clinical data, including electrocardiography, biomarker elevation, and the results of cardiac testing [7]. Figure 2 depicts this expanded capture of patients by APPROACH.

APPROACH has transitioned to a web-based platform over the years, and now provides a more efficient means to support scaling the data collection and reporting across multiple participating hospital sites. This has facilitated the expansion of the initiative beyond the initial participating sites that perform cardiac catheterization. Figure 3 highlights the geographical timeline for APPROACH.

The data collection tools and procedures developed in Alberta by APPROACH have been expanded for use in other provinces; however, because data privacy, protection, and custodianship occur at the provincial level in Canada, the research and data linkage opportunities described in this paper apply only to Alberta. Nevertheless, similar (and parallel) data linkages for research using provincial cardiac registry data have been successfully completed in other provinces in Canada [8, 9].

## Linkage with Administrative Data

Canada has a publicly funded universal health insurance system, with the vast majority of Canadians enrolled [10]. As a result, Canadian administrative data sources contains information on almost the entire population. Further, each patient has a unique personal health number (PHN), allowing patients to be easily identifiable across multiple available databases [11]. These databases also contain information over many years, allowing researchers to follow patients over time. In Canada there are many administrative health care databases. Examples include the Discharge Abstract Database (DAD), the National Ambulatory Care System (NACRS), prescription drug data, and many others [12, 13].

The DAD contains demographic and clinical information on separations from all acute care facilities. Separations include discharges to the community, deaths, patients who sign-out against medical advice, and transfers to other facilities. DAD includes diagnostic, intervention, and patient demographic information and information on patient diagnosis and interventions. NACRS data, meanwhile, contain similar information for outpatient data.

Published outcomes research in health care often rely on administrative databases with limited clinical information about patients. Multivariable risk adjustment based on administrative data is therefore constrained from the outset by the lack of detail on important prognostic factors. An advantage of linkage between clinical and administrative databases is the potential to achieve an increased granularity of prognostic factors. Observational outcome analyses appear frequently in the health research literature. For such analyses, clinical registries are preferred to administrative databases [3]. Missing data, however, are a common problem in any clinical registry, and pose a threat to the validity of observational outcomes analyses.

We previously published on a data merging method for dealing with missing data [14, 15] that involves linking the prospectively-derived cardiac registry data on a patient-by-patient basis to corresponding administrative data (DAD and NACRS), followed by a process of mapping the specific clinical diagnoses present in both the registry data and administrative data to create a single ‘final’ record of baseline diagnoses present for a given patient (Figure 4). Advantages of the methodology that we developed and validated are that it is conceptually simple, it can be readily implemented in many jurisdictions, it can be applied to non-random missing data situations such as ours, and it produces a ‘complete’ dataset that can then be used in subsequent statistical procedures for which data completeness is crucial. This merging method is applied annually in our data cleaning process. Data quality has been a priority within APPROACH over the years and this merging step helps us to ascertain the amount of missing data and query the causes for missing data fields. Using this method, we are able to link 98% of our records and approximately 50% of those cases are enhanced with our merging method. In later work, we demonstrated that strong prediction was achieved using our data enhancement method [16] and that caution is needed in interpreting reports on access to care that use sparsely detailed clinical data sources [17].

Quite apart from data merging to deal with missing data, DAD and NACRS data are also valuable resources to determine follow up utilization of services. We have been able to link with longitudinal data to determine readmission rates and follow up health events of specific interest [18-20]. In addition to DAD and NACRS, APPROACH has also established an annual linkage with Alberta Vital Statistics data for long-term follow up of province-wide data on all-cause mortality. The APPROACH registry has an approved privacy impact assessment. The University of Calgary and University of Alberta Research Ethics Boards have approved APPROACH registry data collection and linkages with these secondary sources.

## Environment for access to other data sources in Alberta

The Canadian Institutes of Health Research (CIHR) have funded a five-year, $48 million provincial-federal partnership (2015-2020) linked to the agency’s Strategy for Patient Oriented Research (SPOR). Within SPOR, CIHR has made core infrastructure investments through the ‘Support for People and Patient-Oriented Research and Trials (SUPPORT) Units’ across Canada, which are locally accessible, multidisciplinary clusters of specialized research resources, policy knowledge, and patient perspective [21].

The mandate of SUPPORT Units is, first and foremost, to enhance the extent to which patients and families are engaged in patient-oriented research initiatives, and also the extent to which impactful knowledge translation is occurring to catalyze meaningful research that positively impacts health. Under this mandate, the Units also aim to assist decision makers and investigators to optimally identify and design research studies, conduct biostatistical analyses, manage data, provide and teach project management skills, and ensure studies meet regulatory standards. The Units advance methods and training in comparative effectiveness research and develop the next generation of methodologists, and provide timely access to data including linked datasets and integrate existing or new databases. Of particular relevance to APPROACH, the SPOR SUPPORT units have a mandate to enable data systems and to support linkage among data systems to accelerate the conduct of clinical and health services research. In Alberta, Alberta Innovates, a provincial funding agency, jointly funds the Alberta SPOR SUPPORT Unit along with CIHR [22]. On a related note, provincial organizations in Alberta have come together to create The Secondary Use Data Project (SUDP), a multi-partner project that is facilitating the enhanced and advanced use of secondary use health and social data for the health and socioeconomic benefit of Albertans. [23]. Along with SPOR Data Platform collaboration, access to data from the province and other sources and data agreements with repository owners has been centralized, of great benefit to the research community [22]. In this enabling context, APPROACH has effectively participated in a number of secondary data use initiatives linked APPROACH data, under the custodianship of AHS, to multiple other data sources, for initiatives led by investigators from across Alberta and other parts of Canada as well. 

## Examples of linkages to other sources

APPROACH investigators and collaborators have published many research studies where the clinically-collected data were linked to other data sources in addition to administrative data. Linking to other sources adds value to the existing information assets and aids in contributing to medical and scientific knowledge. Over 40 graduate and post-graduate trainees, from medicine, nursing, epidemiology, biostatistics, health economics, geography, and the social sciences have used APPROACH’s data in graduate thesis research and contributed to ongoing productivity. Further, the APPROACH information system has been the core source of data for over 200 scientific publications.

### Other clinical registries and databases

Patients with comorbidities have potential to benefit from revascularization, but many are also perceived to be at higher risk of complications and poor long-term outcomes, which may influence treatment decisions and care. APPROACH has allowed teams to explore the care delivered and outcomes of patients with additional phenotypic information provided through linking to other data sources. For example linkage of APPROACH with data collected by the Alberta Kidney Disease Network (AKDN) database has been used to examine selection of cardiovascular procedures and outcomes according to stages of kidney disease as well as to characterize longitudinal changes in kidney function associated with cardiovascular procedures [24—26]. Secondary prevention of coronary artery disease is also an important aspect of cardiovascular care, and understanding what happens to patients after they leave the hospital provides an opportunity to improve long-term care and outcomes. Through data linkage of the APPROACH registry to databases recording patients who were referred to, attended, and completed cardiac rehabilitation programs, collaborative research between APPROACH and cardiac rehabilitation programs has demonstrated the value of and barriers to cardiac rehabilitation after hospitalization with a cardiovascular event [27, 28].

### Laboratory databases

Large quantities of laboratory data, including repeated measures, are available through laboratory information systems (LISs) throughout the province of Alberta, and can be retrieved and linked to provide information that was not originally collected in the registry. Pine et. al. demonstrated that laboratory data can be linked to traditional administrative data to enhance health services analyses focusing on prediction of mortality [29]. Laboratory services in Calgary (one of 3 APPROACH centres in Alberta) are centrally coordinated through a consolidated service delivery model consisting of: one centralized laboratory, specialty testing laboratories, five rapid response laboratories, 19 strategically located community collection sites, a mobile collections service, one centralized transportation system, and one region-wide information system. [30]. This compilation of standardized laboratory data therefore provides the opportunity to link APPROACH for individuals receiving care from all hospitals in Calgary. A subset of common laboratory test data from other provincial LISs is also accessible through a provincially-curated repository. All provincial laboratory data will be even more readily accessible in the near future, when a common provincial LIS is adopted. Members of our team have been able to study the prognostic value of different laboratory values over established risk factors for risk adjustment analyses using these data linkage opportunities [31].

### Geospatial data sources

By utilizing geographic information, we have been able to show the potential utility of geographic information systems (GIS) analysis to inform strategic decisions about the infrastructure of our health care system [32—34]. Socio-economic status data obtained through the federal census, available at geographic level through Statistics Canada [35] also allow us to study differences in access to care for different socio-economic strata. Our analyses suggest that factors other than insurance status and/or ability to pay are at play in access to cardiac care [36]. In conjunction with geospatial data we have been able to link to local publicly available open access geographic data sources. In Alberta’s two major cities, Edmonton and Calgary, we linked data for patients admitted with ACS to geospatial data. Each patient was geocoded to a neighbourhood of residence using their address from hospital records. Crime rates were collected using publicly available data from each city’s police service. Neighbourhood characteristics were gathered from the 2011 Canadian census. This application of data linkage allowed a population-based investigation of the relationship between neighbourhood crime level and incidence of ACS. [36, 37]

### Ongoing linkage projects

Current APPROACH work is focusing on linking APPROACH to ECG data. The MUSE ® (GE Healthcare) system has been used for acquisition, analysis and archiving of ECG data in the Calgary region since 1998. It is now possible to link the longitudinal ECG record in MUSE with APPROACH, further enriching the level of clinical detail available, and permitting a thorough investigation of the evolution of conduction abnormalities over time and their relationships with cardiovascular outcomes. This linkage provides a unique opportunity to describe the natural progression and outcomes of conduction abnormalities in a large population with a substantial period of follow-up. Further, this linkage will serve to demonstrate the feasibility of linking the APPROACH, MUSE and administrative data sets to provide a powerful tool and platform for future research [38].

Another linkage opportunity in Alberta is the ability to link to pharmacy claim data via the Pharmaceutical Information Network (PIN). PIN is a province wide computerized pharmacy/pharmaceutical network that collects key information from physician offices and pharmacies across Alberta and creates a medication profile for each patient. This tool captures >96% of prescriptions for Alberta residents from community pharmacies across the province of Alberta. APPROACH is using these data to characterize long-term patterns of medication use based on outpatient prescriptions, to characterize potential treatment gaps and to conduct pharmacoepidemiology research that leverages the provincial PIN data [39].

Figure 5 summarizes the many different types of data linkages that have been achieved over time with the APPROACH database.

## Impacts of Data Linkage

In addition to over 200 publications, APPROACH has made several methodological contributions to the literature [14—16], helped to develop nationally standardized data definitions [7], secured several competitive research funding awards, and been recognized by the Canadian Institutes of Health Research as a notable health research platform on a national landscape, as mentioned earlier [3]. Furthermore, the initiative’s lead investigator, Dr. Merril Knudtson, an interventional cardiologist and a Canadian leader in cardiovascular outcomes research, was awarded the Order of Canada in 2012 (to recognize a lifetime of outstanding achievement, dedication to the community and service to the nation).

Through data linkage we have been able to study a breadth of conditions. Our linkage to mortality and repeat procedures has enabled extended follow up studies of patients. With additional data sources now being linked, we are able to study hospital readmissions for reasons than cardiovascular disease, which has led to collaborations with researchers in other fields (e.g. oncology, mental health).

## A Future Vision for APPROACH Data Linkage

APPROACH began as an Alberta-wide, collaborative, prospective data collection initiative. The project is now producing detailed clinical information on processes and outcomes of cardiac care in all patients undergoing cardiac catheterization in Alberta. With several national centres implementing the APPROACH system for prospective data collection there is growing potential for leveraging the national distribution of this registry system. Each province’s data elements can be linked and combined to study care at the national level, and to draw provincial comparisons on care and outcomes [40—43]. The APPROACH team in Alberta hosts an annual 2-day national meeting where national and international experts and APPROACH researchers share updates in various domains of health services research, product updates, and development. In partnership with both the CIHI and the CCS, APPROACH has contributed to the development of pan-Canadian data definitions and QIs for evaluating cardiovascular care in Canada [5]. The APPROACH initiative continues to be an important contributor to national efforts to improve data sources on cardiovascular care in Canada.

We have shared the history, current state, and future plans for APPROACH as a generic template of potential interest to other jurisdictions and/or clinical groups. We are in a new era of health information and data science. The future of health is inextricably linked to the future of data and health information initiatives.

## Figures

**Figure 1: Data modules in the Alberta Provincial Project for Outcome Assessment in Coronary Heart Disease (APPROACH) Database:**
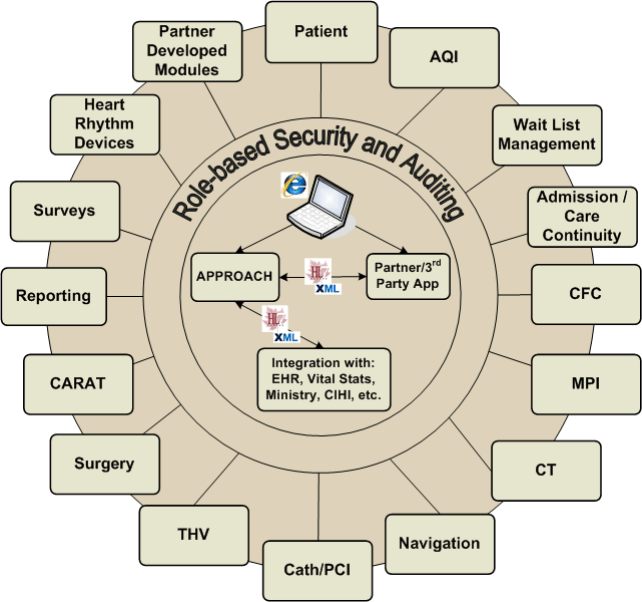


**Figure 2: The scope of the Alberta Provincial Project for Outcome Assessment in Coronary Heart Disease (APPROACH) database – what it captures (enclosed within dotted lines) and what it does not capture. CABG Coronary artery bypass graft; PCI Percutaneous coronary intervention:**
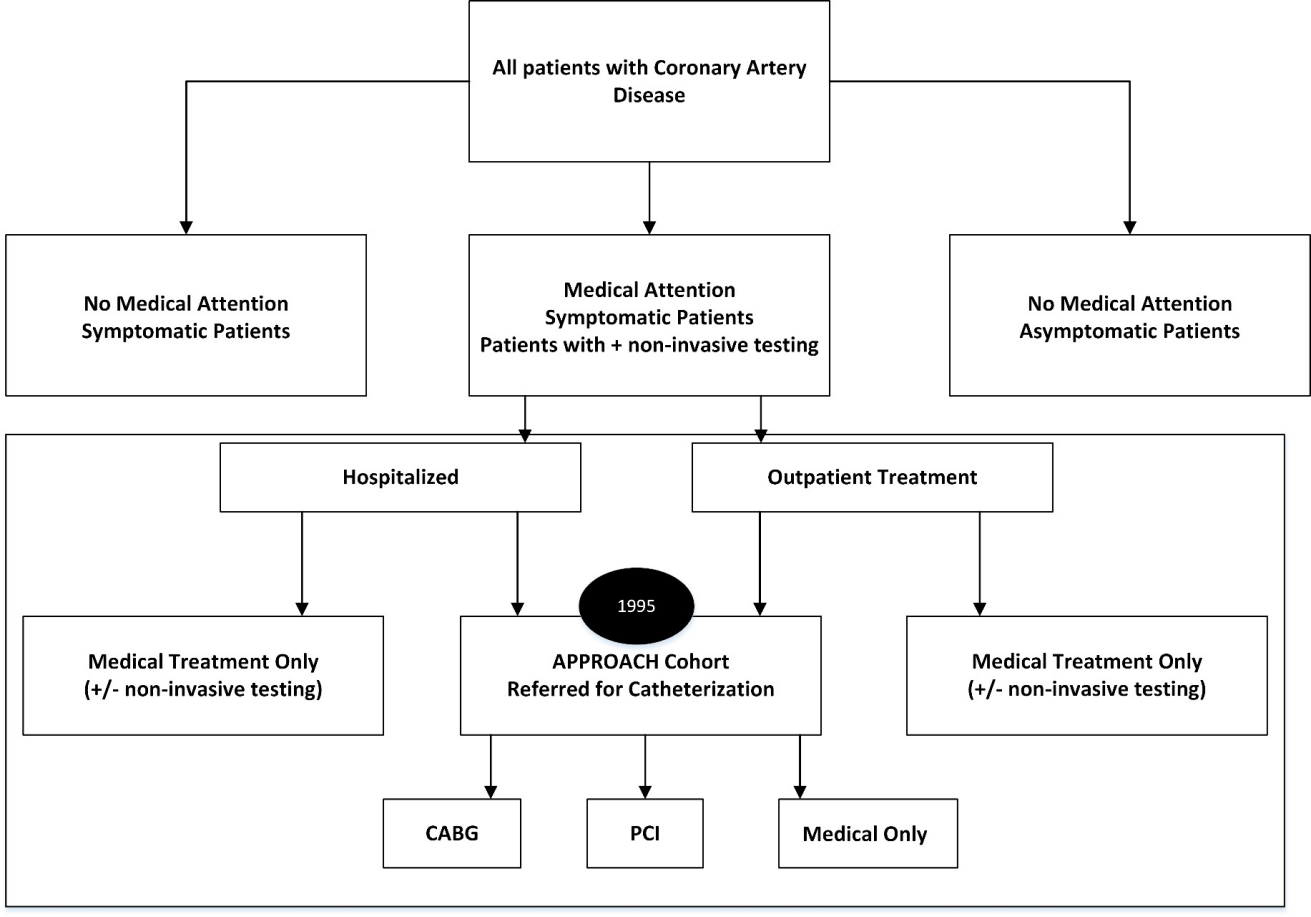


**Figure 3: Geographic timeline of the Alberta Provincial Project for Outcome Assessment in Coronary Heart Disease (APPROACH):**
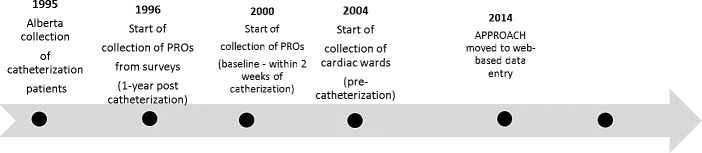

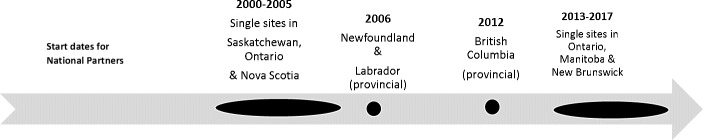


**Figure 4: Merging Process:**
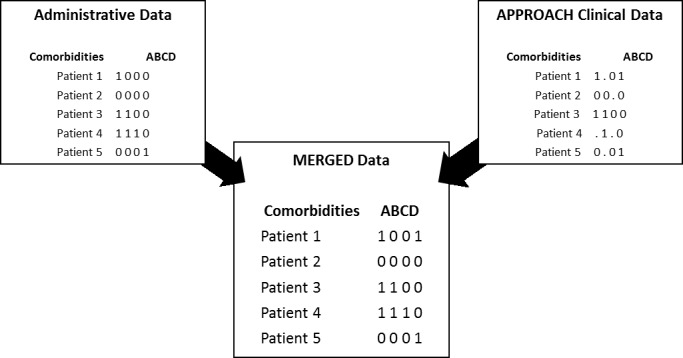


**Figure 5: Data sources to which APPROACH clinical registry is linkable:**
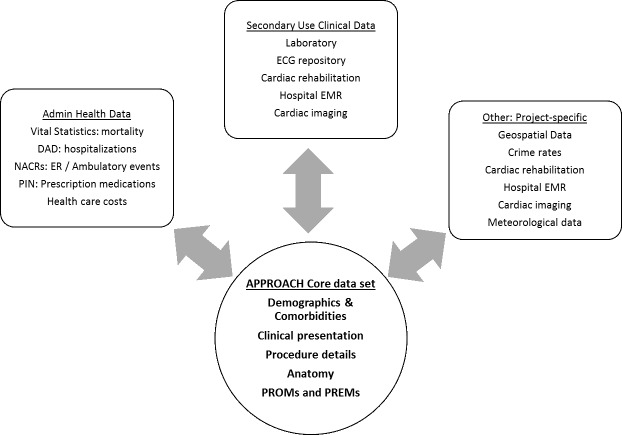
(DAD: Discharge Abstract Data; NACRS: National Ambulatory Care Reporting System; ER: Emergency Room; PIN: Pharmaceutical Information Network; ECG: Electrocardiogram; EMR: Electronic Medical Record)
